# Reply: Evolutionary game theory: lessons and limitations, a cancer perspective

**DOI:** 10.1038/sj.bjc.6605445

**Published:** 2009-11-17

**Authors:** D Dingli, F A C C Chalub, F C Santos, S Van Segbroeck, J M Pacheco

**Affiliations:** 1 Division of Hematology, Mayo Clinic, College of Medicine, Rochester, MN 55905, USA; 2 Universidade Nova de Lisboa, Caparica, Portugal; 3 Université Libre de Bruxelle, Brussels, Belgium; 4 Vrije Universiteit Brussel, Brussels, Belgium; 5 Universidade do Minho, Braga, Portugal

**Sir,**

We read with great interest Dr McEvoy's comments on our paper. His attempt to ‘give a simplified explanation of the underlying concepts to a non-mathematical physician’ resulted in an over-simplification, which we would like to clarify from the start. Strictly speaking, our manuscript discusses multiple myeloma (MM) as an ‘evolutionary game’ between three interacting cell types, which are viewed as different strategies of a cell population. In reducing the interplay of cell lineages to hawks and doves, in whatever variant of the game, Dr McEvoy is pictorially moving along the edges of the simplex (triangles) in Figures 2–4, which, in our view, is too much of a simplification, given that MM development proceeds *across* the simplex, as explicitly shown in our paper. Nonetheless, along the edges of the triangle, hawks and doves play a coexistence game, never a prisoner's dilemma. This is an important point, as most of the evolutionary thinking in cancer has been considered as a simplified version of a prisoner's dilemma between two cell lineages, something that is explicitly abandoned in our work.

Whenever reproduction, mutation and selection occur, evolution is a natural consequence (Cairns, 1975; Tomlinson and Bodmer, 1999). Viewed in this way, cancer is an evolutionary process, albeit an undesirable one with respect to the host. Cancer is clearly a problem of multicellularity and an almost inevitable outcome if an organism is large enough, as what matters is the population of cells at risk, assuming that the organism lives long enough (Lopes *et al*, 2007). However, evolution is a blind process – it is not driven by any specific purpose; cells randomly explore the fitness landscape and the environment selects for the clone with the higher fitness. Indeed, reproductive fitness can only be defined in the context of the environment that selects for or against it. We agree that to date, it has been difficult to experimentally determine the fitness (or, in our case equivalently, the payoff) of mutant cells in a given environment, but it is also true that many experimentalists do not think in terms of evolutionary dynamics. Perhaps one of the benefits of our work is to illustrate the importance of thinking in dynamic terms to better understand cancer and how best to treat it. It is often the case that theory directs experiment – and our work should be considered in this light. Indeed, we believe it is a matter of time before the ‘real’ values of the relevant parameters in such a game can be defined. However, we wish to point out that we mathematically proved that our conclusions hold true for *β*>1 and/or *δ*⩾0. These values are compatible with the known biology of myeloma and the outcome will not change because their absolute values will differ from the ones that we chose to illustrate these dynamics. One of the strengths of Evolutionary Game Theory (EGT) is the generality of its conclusions (Hofbauer and Sigmund, 1998). Furthermore, and in contrast with most previous work on somatic evolution of cancer, here the fitness of a given cell depends on the relative number – the frequency – of cells of that lineage in the population. Again, here the environment has an important role, which has been often neglected in the past.

As we pointed out explicitly in the paper, the EGT that we used is mathematically equivalent to the Lotka–Volterra equations of ecology (Hofbauer and Sigmund, 1998). We trust Dr McEvoy will agree with us that cancer is clearly an ecological problem, certainly at a near-microscopic scale – but nonetheless an ecological problem as such, and ecology makes no use of rationality. Indeed, one of the central results of EGT is that its dynamical formulation – as opposed to the static formulation of Game Theory (GT), which made it so popular in economics and affects all our policies as we speak – does not rely on any argument of rationality (Hofbauer and Sigmund, 1998). It is, however, true, that if a population of irrational cells engaging in some (genetically determined) strategy (they did not choose) evolves towards an Evolutionary Stable State (ESS) (if it exists), the final state will coincide with the strict Nash equilibrium that a pair of rational payoff maximisers will choose when they interact once according to a game with the same payoff matrix. Does this make cells rational? Not at all, and we thank Dr McEvoy's comment, for it illustrates a common confusion that extends well beyond medicine.

Cancer cells have a fitness advantage owing to their mutation profile (Cairns, 1975; Tomlinson and Bodmer, 1999; Beerenwinkel *et al*, 2007). In our model, we do not consider additional mutations that change this profile, as this would mean introducing new strategies in the cell population. Their fitness, however, does not result solely from their mutation profile – it is also dependent on their microenvironment. We purposefully chose MM because its interactions with osteoclasts and osteoblasts are well defined and the general response of the three cell populations to the exchange of cytokines is well accepted. Of course, similar principles can be readily generalised and applied to other tumours and even non-malignant disorders.

We disagree with Dr McEvoy that ‘any ESS reached by treatment can only at best achieve an ESS where normal cells coexist with malignant cells’. In fact, Figure 2b shows precisely that this need not be the case, although at present the cure scenario, although possible, is not under our control. Hence, although it may be difficult to cure a tumour, this depends on where the populations lie with respect to the saddle point (unstable equilibrium) as shown in Figure 2b. If therapies can alter the values of the interacting parameters such that the patient reaches a state to the left of this equilibrium point, in the absence of further mutations, natural selection *will* eliminate the malignant clone, although this may take time. Therapies that can reduce the fitness of malignant cells compared with their normal counterparts exist, with imatinib (and other tyrosine kinase inhibitors, such as dasatinib or nilotinib) being perhaps the best example. Indeed, we have shown that this is the reason for its well-known efficacy in chronic myeloid leukaemia (Dingli *et al*, 2008). We believe it is only a matter of time before other tumours can be treated in a similar manner. Furthermore, reducing the fitness of a cell does not imply reversing the process of carcinogenesis. For instance, the reversible nature of imatinib means that, once treatment is stopped, the disease *may* relapse. Consequently, imatinib does not eliminate *bcr–abl* oncoprotein expression but abrogates its function and therefore reduces the relative fitness of cancer cells.

As a final remark, we would like to point out that our results suggest that the path to win the ‘war on cancer’ is perhaps not to fulfil the goal pictured in the cover of the *Economist* in September 2008, in which all cancers cells must be targeted for elimination. Instead, we may look for our allies in the right place and at the right scale, which, with a small yet intelligent push from our side, may do the job much better, by taking advantage of the same ruthless power of evolution that favoured cancer in the start of the process.
